# N-Cadherin Distinguishes Intrahepatic Cholangiocarcinoma from Liver Metastases of Ductal Adenocarcinoma of the Pancreas

**DOI:** 10.3390/cancers14133091

**Published:** 2022-06-23

**Authors:** Tiemo S. Gerber, Benjamin Goeppert, Anne Hausen, Hagen R. Witzel, Fabian Bartsch, Mario Schindeldecker, Lisa-Katharina Gröger, Dirk A. Ridder, Oscar Cahyadi, Irene Esposito, Matthias M. Gaida, Peter Schirmacher, Peter R. Galle, Hauke Lang, Wilfried Roth, Beate K. Straub

**Affiliations:** 1 Institute of Pathology, University Medical Center of the Johannes Gutenberg-University Mainz, 55131 Mainz, Germany; tiemo.gerber@unimedizin-mainz.de (T.S.G.); anne.hausen@unimedizin-mainz.de (A.H.); hagen.witzel@unimedizin-mainz.de (H.R.W.); mario.schindeldecker@unimedizin-mainz.de (M.S.); dirk.ridder@unimedizin-mainz.de (D.A.R.); matthias.gaida@unimedizin-mainz.de (M.M.G.); wilfried.roth@unimedizin-mainz.de (W.R.); 2 Institute of Pathology and Neuropathology, RKH Klinikum Ludwigsburg, 71640 Ludwigsburg, Germany; benjamin.goeppert@rkh-gesundheit.de (B.G.); peter.schirmacher@med.uni-heidelberg.de (P.S.); 3 Department of General, Visceral and Transplant Surgery, University Medical Center of the Johannes Gutenberg-University Mainz, 55131 Mainz, Germany; fabian.bartsch@unimedizin-mainz.de (F.B.); lisa-katharina.heuft@unimedizin-mainz.de (L.-K.G.); hauke.lang@unimedizin-mainz.de (H.L.); 4 Tissue Biobank, University Medical Center of the Johannes Gutenberg-University Mainz, 55131 Mainz, Germany; 5 Institute of Pathology, University of Heidelberg, 69120 Heidelberg, Germany; o.cahyadi@klinikum-bochum.de; 6 Institute of Pathology, University Clinic Düsseldorf, 40225 Düsseldorf, Germany; irene.esposito@med.uni-duesseldorf.de; 7 Department of Medicine I, University Medical Center of the Johannes Gutenberg-University Mainz, 55131 Mainz, Germany; peter.galle@unimedizin-mainz.de

**Keywords:** liver cancer, cell–cell contacts, adherens junctions, biliary tract cancer, differential diagnosis, tumor biology

## Abstract

**Simple Summary:**

The differential diagnosis of primary liver cancer versus liver metastases of an extrahepatic primary tumor may be challenging, especially in carcinoma of the pancreatobiliary phenotype, as in intrahepatic cholangiocarcinoma or metastases of ductal adenocarcinoma of the pancreas. Nevertheless, the distinction is of fundamental importance for therapy planning. In the frame of this study, we focus on the differential expression and localization of the cell–cell contact-proteins E-cadherin and N-cadherin, markers for cell differentiation in normal epithelia of the pancreaticobiliary tract as well as in derived tumors, and demonstrate that N-cadherin positivity distinguishes intrahepatic cholangiocarcinoma from liver metastases of ductal adenocarcinoma of the pancreas. We propose that N-cadherin-positivity together with other biliary markers may be used for this important histopathological differential diagnosis and may thus improve the accuracy of cholangiocarcinoma diagnosis.

**Abstract:**

Carcinomas of the pancreatobiliary system confer an especially unfavorable prognosis. The differential diagnosis of intrahepatic cholangiocarcinoma (iCCA) and its subtypes versus liver metastasis of ductal adenocarcinoma of the pancreas (PDAC) is clinically important to allow the best possible therapy. We could previously show that E-cadherin and N-cadherin, transmembrane glycoproteins of adherens junctions, are characteristic features of hepatocytes and cholangiocytes. We therefore analyzed E-cadherin and N-cadherin in the embryonally related epithelia of the bile duct and pancreas, as well as in 312 iCCAs, 513 carcinomas of the extrahepatic bile ducts, 228 gallbladder carcinomas, 131 PDACs, and precursor lesions, with immunohistochemistry combined with image analysis, fluorescence microscopy, and immunoblots. In the physiological liver, N-cadherin colocalizes with E-cadherin in small intrahepatic bile ducts, whereas larger bile ducts and pancreatic ducts are positive for E-cadherin but contain decreasing amounts of N-cadherin. N-cadherin was highly expressed in most iCCAs, whereas in PDACs, N-cadherin was negative or only faintly expressed. E- and N-cadherin expression in tumors of the pancreaticobiliary tract recapitulate their expression in their normal tissue counterparts. N-cadherin is a helpful marker for the differential diagnosis between iCCA and PDAC, with a specificity of 96% and a sensitivity of 67% for small duct iCCAs and 50% for large duct iCCAs.

## 1. Introduction

Adenocarcinomas of the pancreatobiliary tract constitute a group of underestimated carcinomas with a devastating prognosis: although tumors of the liver and pancreas account only for the sixth and twelfth most frequent tumors, they present the third and seventh most deadly tumors worldwide [1,2]. Patients with pancreatic ductal adenocarcinoma (PDAC), the most frequent tumor of the pancreas, have a poor one-year survival rate of only 18% for all stages of the disease [3]. Intrahepatic cholangiocarcinoma (iCCA) is the second most common primary hepatic malignancy after hepatocellular carcinoma (HCC), with increasing incidence and yet unfavorable prognosis between that of HCC and PDAC [4,5,6]. In patients without metastatic spread, PDAC and iCCA are both treated with locally curative resection. Metastatic disease is usually treated with chemotherapy [3,4,5]. However, PDAC and iCCA need different chemotherapy regimens. For example, while 5-FU is not the first choice for therapy of PDAC, it is a more common choice for iCCA. As already stated by Lok et al., the differential diagnosis of PDAC metastases and iCCA based on histomorphological criteria alone is challenging [7]. However, there is still no sufficiently specific immunohistochemical marker for this difficult diagnostic dilemma. Therefore, it is pivotal to improve our knowledge of underlying circumstances leading to iCCA and PDAC, unravel potential subgroups, and thereby help improve therapy.

According to the current 2019 WHO classification [8], iCCA are classified into small duct and large duct iCCAs. Depending on their location, extrahepatic cholangiocarcinomas are subdivided into perihilar (pCCA) and distal (dCCA) cholangiocarcinomas [9,10]. Due to their common embryonal origin, in conventional histology, the differential diagnosis of biliary tract carcinoma (BTC) and metastatic PDAC involving the liver may be problematic [11,12].

Cell–cell contacts of adherens junctions are mechanical tethers of multicellular organisms that are required for proper cellular function, embryogenesis, cell differentiation, and maintenance of tissue integrity [13]. Due to their importance in regulating the invasion and metastasis of malignant tumors, they have been extensively studied in tumorigenesis, especially carcinogenesis [14]. The transmembrane domain of adherens junctions is formed by proteins of the cadherin superfamily, Ca^2+^-dependent membrane glycoproteins comprising more than 100 members in humans [15]. The extracellular domains of cadherins connect one cell to the extracellular domain of another cadherin, usually in a homotypic fashion, whereas their intracellular domains are linked via (α-, β-, γ-) catenins to the actin cytoskeleton [16,17]. Cadherin–catenin interaction, especially via the nuclear translocation of β-catenin, influences cell signaling, such as the Wnt-signaling cascade, and plays an important role in the maintenance and homeostasis of polarized epithelial monolayers [18,19,20]. The best-studied cadherin in epithelial cells is E-cadherin (formerly known as uvomorulin and L-CAM [21]), whereas N-cadherin (formerly known as A-CAM) has been predominantly described in mesenchymal and neuroectodermal cells [22,23,24,25]. Loss of E-cadherin in the mouse embryo leads to early death due to defective preimplantation development [26]. Loss of N-cadherin shows defects in the formation of the neural tube and impaired heart development, leading to death on day 10 of mouse development [27]. Due to their cell- and tissue-specific expression patterns, cadherins may be used as immunohistochemical markers to determine the cell of origin, e.g., in malignant tumors. For example, E- and N-cadherin are routinely used in the differential diagnosis of malignant mesothelioma and adenocarcinoma of the lung [28,29]. In addition, loss of E-cadherin is a hallmark of lobular invasive carcinoma of the breast [30] as well as signet cell carcinoma of the stomach [31].

We could previously unravel that hepatocytes harbor equal amounts of E- and N-cadherin and are characterized by a certain type of adherens junction, forming cis-E:N-cadherin heterodimers [32] at the basolateral membrane that are also retained in the respective hepatocellular tumors, such as hepatocellular adenoma and carcinoma. In preliminary analyses, we also detected the colocalization of E- and N-cadherin in small and large bile ducts, so we concluded that the coexpression of E- and N-cadherin may be a characteristic of an endodermal cell lineage, differentiating the liver from other organs, where N-cadherin has only been demonstrated in the process of epithelial-to-mesenchymal transition [32]. In small cohorts of patients, studies by other authors have already demonstrated the presence of N-cadherin, in addition to E-cadherin, in cholangiocarcinomas as well [33,34,35]. To test the utility of E- and N-cadherin in the differential diagnosis of tumors of the pancreatobiliary system, we have analyzed the expression pattern in the physiological biliary tract as well as in a large cohort of patients with iCCA, pCCA, GBC, and PDAC, along with clinical parameters. In addition, we propose a strategy in the workup of these entities that utilizes both commonly available antibodies and N-cadherin.

## 2. Materials and Methods

### 2.1. Tissue Collection

Paraffin-embedded formalin-fixed (FFPE) tissue samples from 1184 patients with iCCA, pCCA, dCCA, GBC, and PDAC, diagnosed at the Institutes of Pathology, University Medical Center Mainz, between 2006 and 2020 and the University of Heidelberg, were collected. Tissue samples were processed in accordance with the regulations of the Tissue Biobank of the University Medical Center Mainz after approval by the local ethics committee (Ethics Committee of the State Medical Council of Rhineland-Palatinate and the University Heidelberg). Tissue microarrays (TMAs) with cores of 2 mm were constructed. For each patient, the primary tumor, corresponding normal tissue, and, if available, precursor lesions, as well as lymph nodes or foreign metastases, were represented. Tumor samples were taken in duplicate, one sample each from central and peripheral tumor areas, to mitigate the impact of intratumoral heterogeneity. Overall, 11,396 TMA cores were evaluated. In addition, twelve cryopreserved tumor samples of iCCA, pCCA, GBC, and PDAC were used for immunoblot analysis and immunofluorescence microscopy. Moreover, cryopreserved normal human, mouse, rat, and bovine liver, gallbladder, and pancreas tissues were used as previously published [32]. Cultured cells of human PDACs were from the lines Capan-1 (ATCC, HTB-79) and Panc-1 (ATCC CRL-1469).

### 2.2. Antibodies and Reagents

Mouse monoclonal antibodies were against E-cadherin (Clone 36, Bioscientia Healthcare GmbH, Ingelheim, Germany), N-cadherin (Clone 32, Bioscientia Healthcare GmbH), and actin (Clone C4, Merck Chemicals GmbH, Darmstadt, Germany). Mouse monoclonal antibodies purchased from Dako Deutschland GmbH, Hamburg, Germany, were against epithelial membrane antigen (EMA; Clone E29), CDX2 (Clone DAK-CDX2), CK7 (Clone OV-TL,), CK20 (Clone K2 20.8), and Ca19-9 (Clone 1116-NS-19-9), as well as Ki-67 (Clone MIB-1). In addition, a rabbit monoclonal antibody was used against E-cadherin (Clone EP700Y, Epitomics, Burlingame, CA, USA), with rabbit polyclonal antibodies against N-cadherin (Calbiochem, Darmstadt; QED Bioscience, San Diego, CA, USA).

### 2.3. Immunofluorescence Microscopy

Cryosections of normal liver and tumor tissues were cut at a thickness of 5 µm, air-dried for 1 h, and fixed with acetone at −20 °C for 10 min. After a permeabilization step for 4 min in 0.1% Triton X-100 and two washing steps in phosphate-buffered saline (PBS), the primary antibodies were applied for 30 min to 1 h, followed by two washing steps for 5 min in PBS and 30 min incubation with the respective secondary antibodies in a humid chamber (cy 3, rabbit, Dianova; Alexa 488 anti-mouse, MoBiTec). After two subsequent washing steps for 5 min in PBS as well as a short washing step in distilled water, the slides were dehydrogenated with 100% ethanol for 5 min and mounted with a DAPI embedding medium. For confocal immunofluorescence microscopy, a laser scanning microscope (LSM 510 Meta; Carl Zeiss AG, Oberkochen, Germany) equipped with Plan Apochromat 63×/1.40 NA oil and Plan-Neofluar 40×/1.30 NA oil objectives was used. AxioVision Release 4.6.3.0 and LSM Image Browser 3.2.0.115 software (Carl Zeiss AG, Oberkochen, Germany) were used for image processing.

### 2.4. Immunoblot Analysis

Cryopreserved human tissue samples were cut into 100–150 5 μm thick sections with a cryostat (Leica CM3050 S) and transferred into tissue homogenizing kit CKMix tubes (Precellys). Afterwards, the tissue sections were supplemented with 500 µl RIPA buffer (50 mM TRIS-HCl, 15 mM EGTA, 100 mM NaCl, 1% (*v*/*v*) Triton X-100, pH = 8.0) and 1:100 Halt™ protease and phosphatase inhibitor (Thermo ScientificTM). The lysis was performed with a Precellys 24 homogenizer at 6500 rpm for 20 s. The homogenate was centrifuged at 12,000× *g* for 5 min at 4 °C, and the pellet was discarded. The protein concentration of the supernatant was determined with the Bradford protein assay (Bio-Rad). The proteins of the total cell lysates were then separated by 8% SDS-PAGE and transferred with a Trans-Blot^®^ TurboTM transfer system (Bio-Rad) onto a nitrocellulose membrane (AmershamTM ProteanTM 0.45 µm NC, GE Healthcare Life science). The membrane was blocked overnight at 4 °C with 1× Tris-buffered saline (A5001, PanReac AppliChem ITW Reagents) supplemented with 0.05% (*v*/*v*) Tween-20 and 5% (*w*/*v*) nonfat dried milk powder (A0830, PanReac AppliChem ITW Reagents). Primary and secondary antibodies were diluted in a blocking solution (1:2000 dilution for E- and N-cadherins and 1:10000 for actin). Gimp software 2.10.28 was used for pixel density analysis of the protein bands. We calculated the E-cadherin/actin and N-cadherin/actin ratios for each band and displayed them relative to the maximum.

### 2.5. Immunohistochemistry

Immunohistochemistry (IHC) was performed on 4 µm thick histological sections of FFPE specimens, according to the manufacturer’s recommendations. Subsequently, slides were digitalized by a whole slide scanner at 40×, with a pixel size of 0.2278 × 0.2278 µm (Nanozoomer, Hamamatsu Photonics, Hamamatsu, Japan).

Specific membranous immunohistochemical stainings for E- and N-cadherin were manually evaluated by scoring the intensity of staining from 0 (absence of staining), 1 (faint membranous staining), and 2 (moderate membranous staining) to 3 (strong membranous staining), as described before [36]. Immunohistochemical staining was performed on tissue specimens present in duplicate, and the mean value was calculated. A staining intensity that had a mean value greater than or equal to 1.5 was assigned to the high expression group, while values less than 1.5 were assigned to the low expression group. The extent of cytoplasmic staining for CK7, CK20, EMA, and CA19-9 and the nuclear staining for CDX2 were classified as negative, weak, moderate, and strong. As a measure of proliferation activity indicative of aggressive tumor behavior, the Ki-67-staining was correlated with E- and N-cadherin expression.

A quantitative assessment of E- and N-cadherin expression levels was undertaken using QuPath version 0.2.3, an open-source software [37]. TMA cores were de-arrayed. Stain vectors and background vectors were individually set in each slide and further analyzed using the “cell detection” algorithm in QuPath. Cellular chromogen 3,3’-diaminobenzidine-tetrahydrochloride-dihydrate mean levels were classified into four categories, using empirical threshold scores (0–0.21 for 0, >0.21 for 1+, >0.45 for 2+, and >0.7 for 3+). These levels provided a relatively low threshold for the discrimination of negative stains (score 0) and faint stains (1+) and a relatively high threshold for the highest achievable score. Ki-67 index estimation was performed after de-arraying and color decomposition using the positive cell detection algorithm in QuPath. The tumor was annotated using a detection classifier. Due to intertumoral heterogeneity, we used custom-tailored classifiers on a case-to-case basis to ensure the proper separation of tumor and non-tumor tissue. This entailed the application of the random trees classifier to train QuPath interactively to distinguish tumor cells from stromal and inflammatory cells. The H-score for E- and N-cadherins was calculated from the extent and intensity of staining, giving scores ranging from 0 to 300 [38]. Ki-67 index estimation was conducted with only one threshold, resulting in a mean percentage of positive tumor cells. TMA cores that could not be evaluated, e.g., due to loss of tissue during staining, were excluded from the study.

### 2.6. Statistics

Statistical analyses were performed using IBM SPSS Statistics Version 27 (IBM Corp., Armonk, NY, USA). To assess the data for normal distribution, the Shapiro–Wilk test was applied for sample sizes below 30. According to the central limit theorem, the distribution above a sample size of 30 is considered normally distributed [39]. An unpaired t-test was used to compare mean H-scores between each tumor entity as well as respective lymph node metastases and normal tissues. Because of different sample sizes, we conducted Hedges’ *g*-test to determine effect size. For non-normally distributed data, we used the Mann–Whitney U-test and calculated the effect size *r*, according to Fritz et al. [40]. To determine the relationship between two variables, we calculated the Spearman rank-order correlation coefficient for non-metric data and the Pearson correlation coefficient for metric and normally distributed data. We assessed the strength of correlation coefficient values according to Chan [41]. Overall survival and recurrence-free survival were identified as the time measured from initial diagnosis to death or cancer recurrence, respectively, and censored at the last clinical follow-up. Using the E-/N-cadherin H-scores from our cohort and the *CDH1* and *CDH2* mRNA data from the TCGA cohort [42], both optimal cut-off values were calculated using the Charité Cutoff Finder [43]. Survival analyses were plotted using the Kaplan–Meier model and compared by log-rank test. For the survival analysis of our cohort, we only used primary iCCA, and we excluded cases with unresectable tumors. To evaluate the discrimination of the dichotomous variables (high vs. low N-cadherin expression), we used a variation of the chi-squared test, McNemar’s test [44]. A *p*-value ≤ 0.05 was considered statistically significant.

## 3. Results

### 3.1. Colocalization of E- and N-Cadherin Characterizes Epithelia of the Physiological Biliary Tract

After we discovered a novel type of adherens junction involving cis-E:N-cadherin heterodimers in the hepatocytes of embryonic and adult liver that also stably characterizes hepatocellular adenoma and carcinoma [32], we were interested in investigating E- and N-cadherin in the embryonically related cells of the biliary tract and pancreas as well as in derived tumors of the pancreatobiliary system.

**Figure 1:**
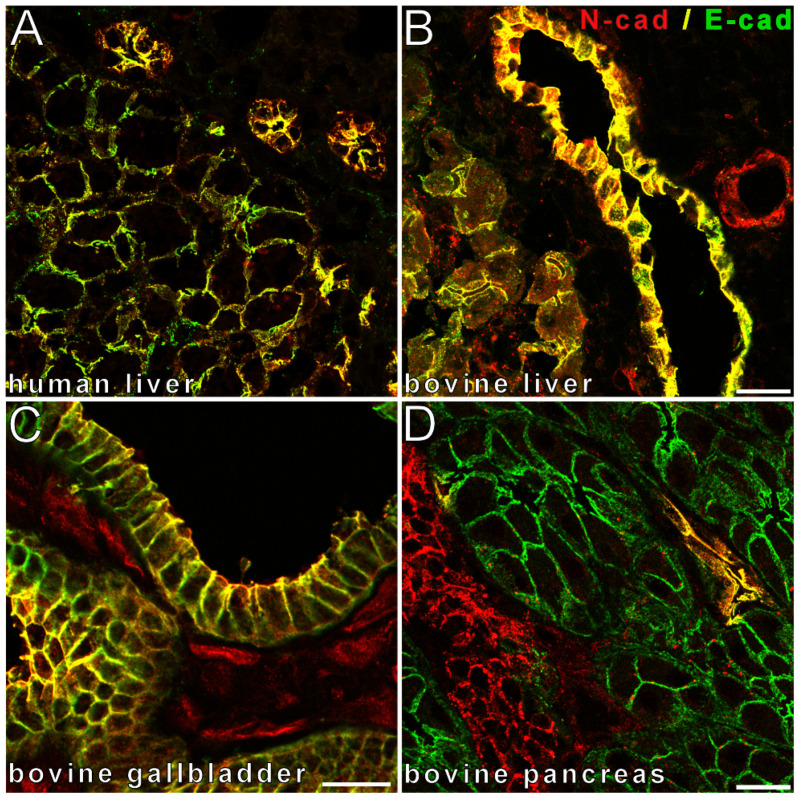
Colocalization of E- and N-cadherin is conserved in normal epithelia of the pancreatobiliary tract. Double-label laser scanning immunofluorescence microscopy shows complete colocalization (resulting in yellow color in the merge images) of E-cadherin (E-cad, Alexa 488, green), with N-cadherin (N-cad, cy3, red) in small bile ducts in normal human liver (**A**), in a large bile duct in normal bovine liver (**B**), as well as in bovine gallbladder epithelium (**C**) and pancreatic duct epithelium in normal bovine pancreas (**D**). Note the colocalization of E- and N-cadherins in normal hepatocytes (**A**,**B**), the positivity of vessels for only N-cadherin but not E-cadherin (**B**,**C**), E-cadherin-positivity with N-cadherin-negativity in pancreas acini (**D**), and differential N- and E-cadherin expression in pancreatic islet cells (**D**). Bar length: 20 µm.

Using cryopreserved mouse, rat, bovine, and human liver tissues, complete colocalization of E- and N-cadherin at the basolateral membrane of hepatocytes was detected, as previously published. Moreover, the epithelial cells of small and large intrahepatic bile ducts also showed complete colocalization at the basolateral membrane, whereas stromal cells of the portal tracts and endothelia of larger vessels were positive for N-cadherin and negative for E-cadherin, as expected. In the gallbladder epithelium, however, variability was observed among different species as rats did not harbor a gallbladder, mouse and human gallbladder epitheliums were positive only for E-cadherin, and bovine gallbladder was positive for both E- and N-cadherin. The extrahepatic bile ducts showed positivity for E-cadherin; however, minor amounts of N-cadherin were observed when compared to intrahepatic bile ducts. In the pancreas, acinar epithelial cells were only positive for E-cadherin, islet cells were positive in part for N-cadherin and in part for E-cadherin, and pancreatic ducts were only positive for E-cadherin, except for bovine pancreas, where pancreatic ducts demonstrated positivity for both E- and N-cadherin (Figure 1). Some larger human pancreatic ducts displayed faint amounts of N-cadherin as well.

**Figure 2:**
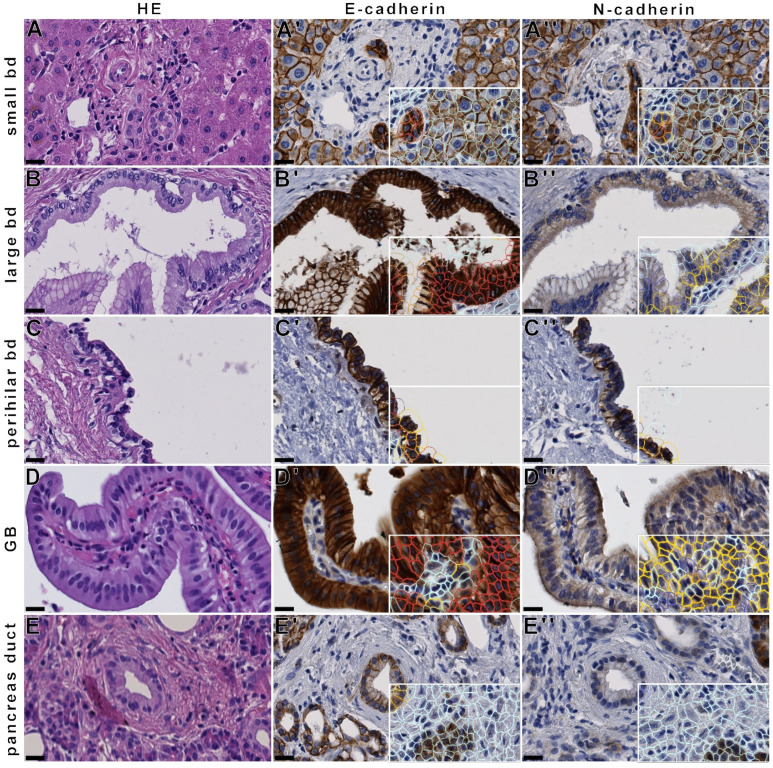
E- and N-cadherin expression in physiological epithelia of the human pancreatobiliary tract. Immunohistochemical analysis of E- and N-cadherins demonstrates strong E-cadherin expression in small (**A**) and large bile ducts (**B**) in human liver, in a perihilar bile duct (**C**), in gallbladder epithelium (**D**) and in pancreas ducts and acini (**E**), whereas N-cadherin expression declines from strong expression in intrahepatic and perihilar bile ducts to faint expression in gall bladder epithelium, with negative expression in pancreatic ducts. QuPath analysis is shown on the bottom right (**A’**–**E’**,**A’’**–**E’’**). (**A’**): manual score 3.0, E-cadherin H-score 268.75; (**A’’**): manual score 3.0, N-cadherin H-score 218.75; (**B’**)**:** manual score 3.0, E-cadherin H-score 268.56; (**B’’**): manual score 1.5, N-cadherin H-score 67.73; (**C’**): manual score 3.0, E-cadherin H-score 212.39; (**C’’**): manual score 2.5, N-cadherin H-score 85.43; (**D’**): manual score 3.0, E-cadherin H-score 290.83; (**D’’**): manual score 2.0, N-cadherin H-score 58.47; (**E’**): manual score 2.5, E-cadherin H-score 105; (**E’’**): manual score 0.5, N-cadherin H-score 8.33. Cells with colored border: white—stromal cells, blue—epithelial cell 0, yellow—epithelial cell 1+, orange—epithelial cell 2+, red—epithelial cell 3+. Black bar: 50 µm (**A**–**E**,**A’**–**E’**,**A’’**–**E’’**) Black bar: 20 µm (**A**–**E**,**A’**–**E’**,**A’’**–**E’’**).

The respective findings were reproduced immunohistochemically in a large collection of human FFPE-material of small and large bile ducts, perihilar bile ducts, gallbladder epithelium, and pancreatic ducts, as evaluated manually as well as with quantitative analysis (Figure 2). In normal pancreatic ducts, using immunohistochemistry, apical secretions are frequently strongly stained by the used N-cadherin antibody that is not observed in immunofluorescence microscopy, resulting in a false-positive N-cadherin H-score, as determined by QuPath (Figure S1).

In summary, during physiological conditions, such as hepatocytes, the epithelia of the intrahepatic bile ducts harbor high amounts of both E- and N-cadherin. N-cadherin expression markedly declines along the biliary tree from intrahepatic bile ducts with a strong expression to extrahepatic bile ducts and pancreatic ducts with faint or no expression.

### 3.2. N-Cadherin Distinguishes Intrahepatic Cholangiocarcinoma from PDAC

**Figure 3:**
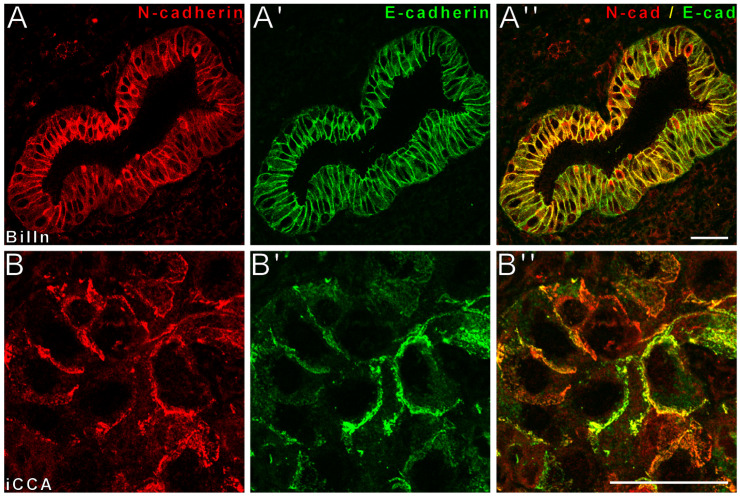
E- and N-cadherin are retained in human BilIN-lesions as well as in iCCA. Double-label laser scanning immunofluorescence microscopy shows partial colocalization of E-cadherin (E-cad, Alexa 488, green) and N-cadherin (N-cad, cy3, red) in BilIN-lesions (**A**–**A’’**) as well as in poorly differentiated iCCA (**B**–**B’’**). Besides complete colocalization, as demonstrated by the merged yellow color, the cell membrane also stretches with more N-cadherin or more E-cadherin, as seen. Bar length: 20 µm.

To analyze whether biliary tumors and precursor lesions recapitulate the same E- and N-cadherin expression pattern as physiologically present in intrahepatic bile ducts, we analyzed biliary intraepithelial neoplasia (BilIN) as well as iCCA. In double-label laser scanning immunofluorescence microscopy, biliary intraepithelial neoplasia and iCCA demonstrated partial to complete colocalization of E- and N-cadherin at the cell membrane (Figure 3).

**Figure 4:**
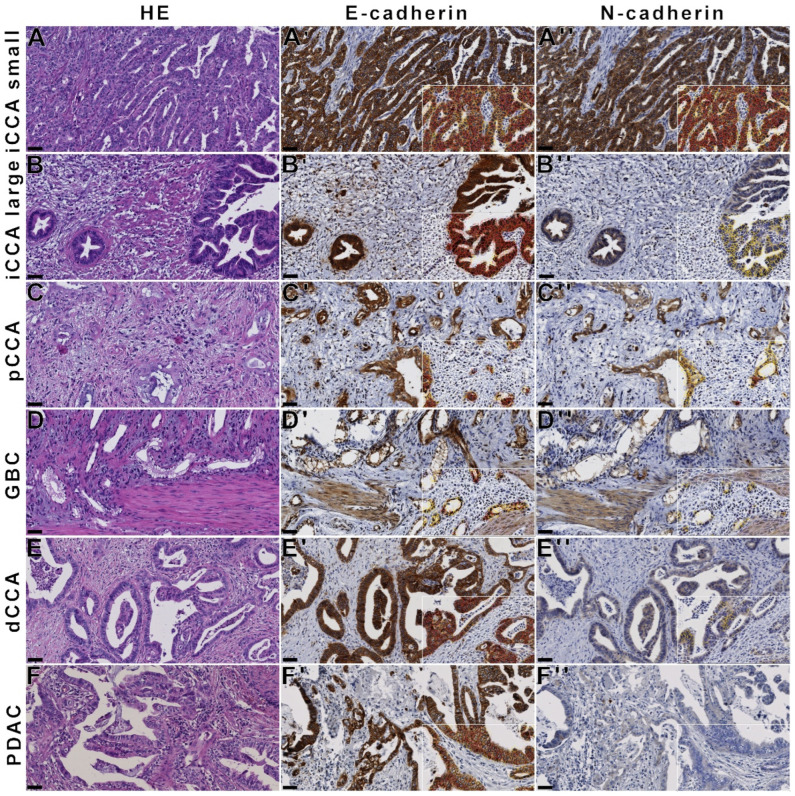
Stable E-cadherin and differential N-cadherin expression in carcinoma of the pancreatobiliary tract. With immunohistochemistry, high N-cadherin expression is detected in iCCA (**A**,**B**) and pCCA (**C**), whereas only faint N-cadherin expression is detected in GBC (**D**) and dCCA (**E**); no N-cadherin is detected in PDAC (**F**)**.** In contrast, E-cadherin is highly expressed in all carcinomas of the pancreatobiliary tract. QuPath analysis on the bottom right (**A’**–**F’**,**A’’**–**F’’**). (**A’’**): manual score 3, N-cadherin H-score 131.24; (**B’’**)**:** manual score 2.5, N-cadherin H-score 80.40; (**C’’**): manual score 2.5, N-cadherin H-score 132.65; (**D’’**): manual score 2.0, N-cadherin H-score 64.18; (**E’’**): manual score 1.5, N-cadherin H-score 46.17; (**F’’**): manual score 1.0, N-cadherin H-score 1.67. Cells with colored border: white—stromal cells, blue—tumor 0, yellow—tumor 1+, orange—tumor 2+, red—tumor 3+. Black bar: 50 µm (**A**–**F**,**A’**–**F’**,**A’’**–**F’’**).

To further analyze E- and N-cadherin expression in a large collection of tumors of the pancreatobiliary tract, together with metastases precursor lesions, in comparison to the respective normal tissues, we used immunohistochemistry for E- and N-cadherin in tissue microarrays comprising 1184 patient samples (Table 1). Nearly every biliary tract cancer and PDAC, as well as their associated lymph node metastases, precursor lesions, and associated non-neoplastic tissue, showed a high expression of E-cadherin. However, N-cadherin expression was strongest and most stable in small duct iCCA, and expression declined in the following order: large duct iCCA, BilIN, pCCA, GBC, dCCA, with the lowest to no expression in PDAC (Figure 4).

Consequently, N-cadherin immunohistochemistry distinguished small and large duct iCCAs from PDACs with a specificity of 96.30% and a sensitivity of 67.01% for small duct iCCAs and a sensitivity of 50.43% for large duct iCCAs (each *p* < 0.01).

To test the practical use of N-cadherin for the differential diagnosis of iCCA to liver metastases of PDAC in liver biopsies taken before therapy planning, we retrospectively analyzed each ten-punch biopsy of clinicopathologically definite iCCA and liver metastases of PDAC and long clinical follow-up. Additionally, in punch biopsies, N-cadherin proved to be a valuable marker to differentiate iCCA and PDAC. Nevertheless, the analysis was complicated by the fact that the original bile ducts, as well as residual hepatocytes enclosed in the tumor, stained strongly positive for N-cadherin.

In addition, we analyzed the known pancreatobiliary tract markers CK7, CK20, CA19-9, EMA, and CDX2 in control (Tables S2–S6). Most BTCs and PDACs showed staining reactions against CK7 and EMA, with negativity against CK20 and CDX2. CA19-9 was weakly expressed in small duct iCCA and showed stronger staining in PDACs. However, the expression was often variable. None of the established markers alone were superior to N-cadherin in the differentiation of iCCA and PDAC.

**Figure 5:**
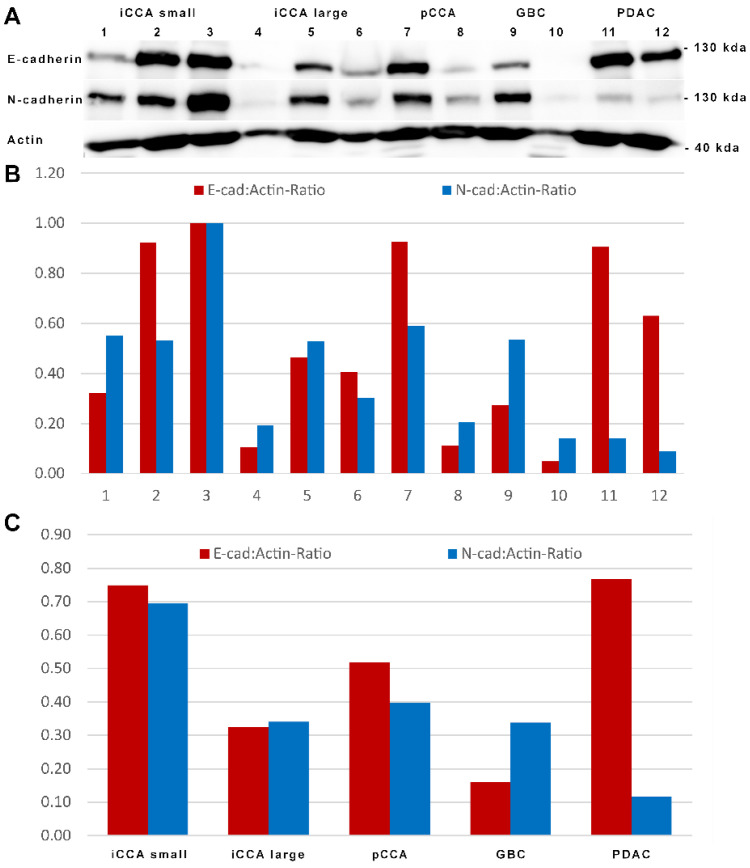
Semiquantitative immunoblot analysis shows equal levels of E- and N-cadherin in iCCA and pCCA, whereas PDAC displays predominant E-cadherin expression. (**A**): Immunoblot analysis of whole tissue lysates of iCCA (small and large duct iCCA (bands 1–3 and 4–6)), pCCA (bands 7 and 8), GBC (bands 9 and 10), and PDAC (bands 11 and 12) was probed with antibodies against E- and N-cadherin. Of note, whole tissue lysates were N-cadherin-positive in stromal cells and vessels. Equal amounts of protein were loaded and equilibrated with actin. Molecular mass markers are depicted on the right side. The uncropped Western blots are shown in Figure S4. Graphical representation of the pixel density analysis of the respective bands (**B**) and the respective tumor (**C**).

The expression pattern of E- and N-cadherin was reproduced in the immunoblot analysis of whole tissue lysates of six cryopreserved iCCAs (three each of the small and large duct types, and two each of pCCA, GBC, and PDAC) (Figure 5).

In whole tissue lysates of iCCA, pCCA, and GBC, E- and N-cadherin were detected in almost equal amounts at about 120 and 130 kDa, respectively. In accordance with our immunohistochemical data, in PDAC, minor amounts of N-cadherin were still detected, possibly due to the N-cadherin in the stromal cells and vessels included in the whole tissue lysates.

To further substantiate the expression pattern of N-cadherin in PDAC, we used cultured PDAC cells of the lines Capan-1 and Panc-1. Again, large amounts of E-cadherin but only small amounts of N-cadherin were detected by immunoblot. N-cadherin staining was observed on membranes of only individual cells by immunofluorescence microscopy, whereas the vast majority of Capan-1 and Panc-1 cells were negative.

In summary, the expression pattern of E- and N-cadherin in pancreatobiliary tumors recapitulates their expression along the normal bile duct tree and pancreatic ducts. Therefore, N-cadherin-positivity may be able to distinguish iCCA from PDAC.

### 3.3. Expression of E- and N-Cadherin in BTC Is Retained in Respective Metastases

Since E- and N-cadherin have generally been implicated in the process of epithelial-to-mesenchymal transition (EMT) during the invasion and metastases of carcinomas, we compared the respective primary carcinomas with their lymph node and foreign metastases and evaluated the intensity of staining by means of a semiquantitative, automated analysis (QuPath). The mean E-cadherin H-scores of primary BTC were significantly higher compared to their lymph node metastases, while in PDAC, we found no respective difference. GBC showed the highest effect (*p* < 0.01; *g* = 1.426), decreasing from dCCA (*p* < 0.01, *r* = 0.33) to iCCA of the small (*p* < 0.01, *r* = 0.25) and large duct type (*p* < 0.01, *r* = 0.23). The mean E-cadherin H-score in the normal tissue was higher than in their respective primary carcinomas, arguing for the downregulation of E-cadherin during carcinogenesis. We found significant effects in GBC (*p* < 0.01; *g* = 0.585), dCCA (*p* < 0.01; *g* = 1.245), iCCA of the large duct type (*p* < 0.01; *g* = 1.376), and pCCA (*p* < 0.01; *g* = 0.748). No significant differences were found for iCCA of the small duct type and PDAC and their respective normal tissues. The mean N-cadherin H-scores of dCCA (*p* < 0.01, *r* = 0.29) and pCCA (*p* < 0.01, *g* = 0.542) were higher in lymph node metastases when compared to their respective primary carcinomas. iCCAs of the large and small duct types, GBC, and PDAC, as well as their associated lymph node metastases, did not show a significant difference. The mean N-cadherin H-score was higher in normal tissue than in associated carcinomas, again arguing for the downregulation of N-cadherin during carcinogenesis. The effect size was highest in PDAC (*p* < 0.01; *g* = 1.071), and it decreased from dCCA (*p* < 0.01; *g* = 0.924) to iCCA of the small duct type (*p* < 0.01; *g* = 0.912), pCCA (*p* < 0.01; *g* = 0.602), and GBC (*p* < 0.01; *g* = 0.439). There was no significant difference for iCCA of the large duct type. Both E- and N-cadherin expression was thereby downregulated during the late stages of carcinogenesis, such as invasion and metastasis formation.

### 3.4. Significance of E- and N-Cadherin for the Prognosis of Patients with Carcinomas of the Pancreatobiliary System

To investigate the prognostic impact of E- and N-cadherin in intrahepatic cholangiocarcinoma, we used the TCGA cohort as an independent cohort (n = 36). In the TCGA cohort, low mRNA expression of both *CDH1* (E-cadherin) and *CDH2* (N-cadherin) was significantly associated with unfavorable outcomes. In addition, an increase in nodal status, T- and UICC-stage, and R-status, as well as vascular invasion, was associated with worse survival.

In all tumors examined in our patient cohort, there was no clear trend between the grading or the Ki-67 proliferation index and the E-cadherin expression (Figure S2), while higher N-cadherin H-scores were weakly correlated with a higher proliferation rate (*r* = 0.207, *p* < 0.01). Concerning cadherin expression in BTC, patients with lower E-cadherin H-scores showed a higher T-stage (ρ = − 0.143, *p* < 0.01). Nevertheless, no correlation was detected between E-cadherin and N-cadherin H-scores and lymphangioinvasion, vascular or perineural invasion, N-stage, grading, and tumor size; there was also no correlation between E-cadherin H-score and proliferation (Ki-67-score) and N-cadherin H-score and T-stage.

In iCCA, the E-cadherin H-score was not associated with a significant difference in recurrence-free survival. In contrast, it was shown that a low N-cadherin score was associated with a poorer prognosis (Figure S3; concerning patient details, see Table S1).

## 4. Discussion

In this study, we demonstrate that E- and N-cadherin are constitutively expressed in cholangiocytes under physiological conditions and retained in BilIN and iCCA in a large clinicopathologic cohort of patients. iCCA and the respective normal bile ducts have already been shown to be positive for N-cadherin, especially in small duct iCCAs [34,35,45,46]. Nevertheless, N-cadherin is only present in minor amounts in physiological extrahepatic bile ducts, as well as in dCCA and pCCA, and is negative or only faintly expressed in physiological pancreatic ducts as well as in PDAC. Thereby, N-cadherin expression in the physiological biliary tract and associated BTC decreases sequentially on a continuous spectrum from small bile ducts and small duct iCCAs to larger and extrahepatic bile ducts and their respective carcinomas. E- and N-cadherin expression in pancreatobiliary carcinomas recapitulates their expression in the respective physiological epithelia of the pancreatobiliary tree, as already illustrated for normal hepatocytes as well as hepatocellular adenoma and carcinoma [32]. Coexpression of E- and N-cadherin may thereby constitute a characteristic of epithelia and derived tumors of the hepatobiliary system. N-cadherin is not restricted to mesenchymal and neuroectodermal cells, as postulated by [23,24,25], but is also constitutively expressed in special endoderm-derived epithelia.

The characteristic expression pattern of N-cadherin in iCCA and its absence in PDAC allow for an immunohistochemical differentiation of these entities. Only one study has previously investigated the usefulness of N-cadherin to discriminate between metastatic PDAC and iCCA—an indicative study with only 31 cholangiocarcinomas [33]. In our study, we obtained comprehensive results in a large and well-characterized clinicopathologic collection of 312 iCCAs as well as 131 PDACs, supporting the use of N-cadherin in this differential diagnosis. Coexpression of E- and N-cadherin may help differentiate the primary liver carcinomas HCC and iCCA from liver metastases originating from an extrahepatic primary tumor. So far, N-cadherin has not been demonstrated in the vast majority of extrahepatic malignancies apart from EMT. Own preliminary results in this respect demonstrated the absence of N-cadherin in most gastrointestinal, tuboovarial, mammary, and lung adenocarcinomas, as well as urothelial carcinomas, so N-cadherin may even help in the differential diagnosis of primary liver cancer from liver metastases of adenocarcinomas of unknown primary tumors. Further studies are underway in this respect. In addition to the N-cadherin analyzed in the frame of this study, albumin RNA in situ hybridization (albumin ISH) may be used in the differential diagnosis between iCCA and metastatic PDAC. However, much like N-cadherin, only in the case of positive expression can albumin ISH guarantee the diagnosis of iCCA [47], so both methods may be used complementarily. Especially in the case of large duct iCCAs, this may hold true as they are positive for albumin RNA ISH in only 18% of cases [48], whereas N-cadherin has a positive rate of 50%.

Of note, loss or downregulation of E-cadherin together with de novo expression or upregulation of N-cadherin has been implicated in the process of EMT in different types of carcinoma and has been correlated to an unfavorable prognosis (the so-called “cadherin switch”) [49,50]. However, the roles of E- and N-cadherin are much more diverse and versatile, which is evident by their controversial roles in different tumors. For example, low expression of N-cadherin is correlated with metastatic dissemination in neuroblastoma [51], and aberrantly high expression of E-cadherin is a hallmark of ovarian carcinoma [52]. Remarkably, the inverse phenomenon of a mesenchymal–epithelial transition (so called MET) has also been described during dedifferentiation in an example of a hematopoietic neoplasm [53]. Many more contradictory and context-dependent roles of different cadherins have been described [54]. Concerning primary liver carcinoma, besides iCCA, HCC also seems to be an exception to the general assumption of EMT as hepatocytes and bile duct epithelial cells contain high amounts of N-cadherin physiologically. Therefore, it is not astonishing to find that in our study, both N- and E-cadherin expression in BTC and PDAC was lower than in the associated normal tissues. Only in associated lymph node metastases was a trend to higher expression of N-cadherin noted; yet, so far, this is of unclear significance. Downregulation of N-cadherin, but not E-cadherin, was significantly associated with poorer outcomes. In our analyses of the independent TCGA cohort, low E-and N-cadherin mRNA levels were both associated with poor survival. Mechanistically, alterations in E-cadherin have been shown to lead to the activation of Wnt signaling and the Hippo signaling pathway. In addition, E-cadherin may interact with epidermal growth factors, the dysregulation resulting in dysmorphogenesis and altered gene expression [55]. In a study by Techasen et al., invasion and migration of cholangiocarcinoma cell lines were increased by siRNA-mediated reduction of E-cadherin levels with the upregulation of vimentin. Furthermore, low immunohistochemical E-cadherin expression was significantly associated with lymph node metastasis. There was no significant association with patient age, sex, histology, and iCCA subtype. [56]. In another study, immunohistochemical examination of iCCA showed a significantly lower expression of E-cadherin than in adjacent tissue and normal bile duct tissue, confirming our results [57]. Reduction of immunohistochemical E-cadherin expression in iCCA compared to normal tissue has been reported in many studies, often correlating with a higher tumor grade [58,59,60,61,62]. We, therefore, suggest that the downregulation of E- and N-cadherins in BTC is a general phenomenon of dedifferentiation, as it is also commonly observed for various other immunohistochemical marker proteins.

In the frame of this study, we could successfully utilize digital image analysis of E- and N-cadherin as membrane-bound markers as it may also prove helpful in the scoring of other immunohistochemical stains. When compared, eyeballing estimation and digital analyses were highly concordant when the digital analysis was adequately trained. Our approach uses manual analysis to assess the expression level and digital analysis to assess the quantitative staining reaction. This should provide a comprehensive assessment and the future use of a manual evaluation in daily routine. Other frequently used membrane biomarkers with relevance for pathology include Her2/neu in breast and stomach cancer, as well as the various cluster of differentiation (CD) antigens in hematopathology. A comprehensive study on punch biopsies for Her2/neu has already shown that manual analysis and digital analysis achieve high concordance rates [63].

To summarize, the expression of N-cadherin in BTC is a characteristic of its biliary tract differentiation and may not reflect EMT.

## 5. Conclusions

**Figure 6:**
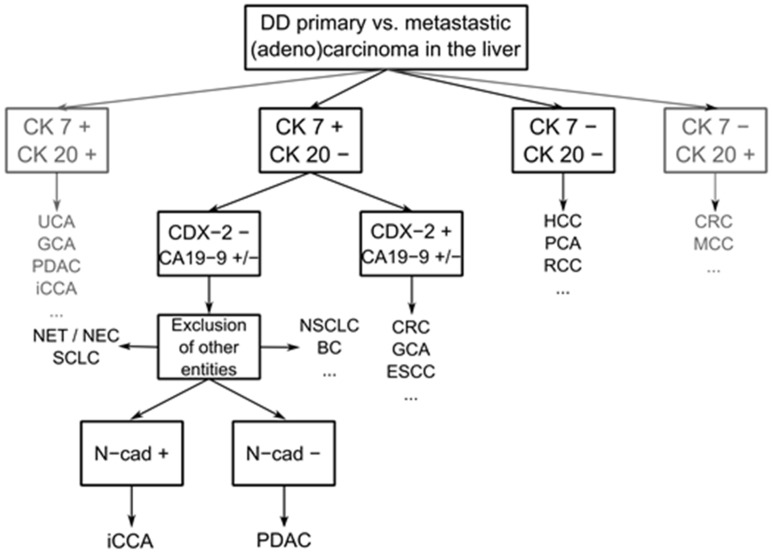
Schematic immunohistochemistry workflow for the differential diagnosis of metastatic and primary adenocarcinomas of the liver. N-cadherin may be used to differentiate the origin of CK7+/CK20− and CDX2-/CA19-9 −/+ pancreatobiliary adenocarcinomas. BC: breast carcinoma. CRC: colorectal carcinoma. ESCC: esophageal squamous carcinoma. GCA: gastric carcinoma. HCC: hepatocellular carcinoma. MCC: Merkel cell carcinoma. NEC: neuroendocrine carcinoma. NET: neuroendocrine tumor. NSCLC: non-small cell lung cancer. PCA: prostate cancer. RCC: renal cell carcinoma. SCLC: small cell lung cancer. UCA: urothelial carcinoma.

In conclusion, in BTC, E- and N-cadherin are expressed in a tissue-specific manner. N-cadherin is a highly specific marker to differentiate iCCA from liver metastases of PDAC. High expression of N-cadherin in adenocarcinomas of pancreatobiliary morphology thereby suggests the diagnosis of iCCA and not PDAC. Moreover, the differential expression of CA19-9 and CK7 versus CDX-2 and CK20 may be used to confirm progeny from the gastrointestinal tract and help diagnose iCCA (Figure 6). We recommend N-cadherin immunohistochemical staining to distinguish biliary origin rather than pancreatic origin.

## Figures and Tables

**Table 1:** Immunohistochemical evaluation of E- andf N-cadherin in iCCA primary carcinoma, premalignant lesions, lymph node metastasis, and normal/ non-neoplastic adult tissue.

			E-Cadherin			N-Cadherin			
Category	Subcategory	Cases	Score	H-Score	High [%] ^†^	Score	H-Score	High [%] ^†^	Ki-67
**Lymph node metastases**	**iCCA small duct type**	18	2.88 ± 0.28	232.59 ± 39.18	100	1.93 ± 0.98	64.06 ± 63.07	64.29	23.02 ± 14.96
	**iCCA large duct type**	15	2.98 ± 0.06	206.04 ± 33.28	100	1.12 ± 0.85	41.78 ± 45.50	33.33	23.01 ± 11.51
	**pCCA**	45	2.90 ± 0.32	226.83 ± 54.26	100	0.79 ± 0.63	36.78 ± 47.16	25.64	21.80 ± 17.08
	**GBC**	34	2.86 ± 0.49	241.85 ± 39.49	96.97	0.47 ± 0.66	13.07 ± 37.30	6.45	26.80 ± 22.63
	**dCCA**	13	3.00 ± 0	259.14 ± 15.95	100	0.73 ± 0.59	34.51 ± 47.32	8.33	24.79 ± 17.76
	**PDAC**	67	2.75 ± 0.53	178.56 ± 62.63	97.01	0.20 ± 0.44	2.09 ± 7.54	3.13	n.a.
**Primary carcinoma**	**iCCA small duct type**	196	2.90 ± 0.29	162.35± 76.25	98.96	1.76 ± 0.92	38.97 ± 53.32	67.01	16.06 ± 16.06
	**iCCA large duct type**	116	2.85 ± 0.32	149.46 ± 72.36	99.13	1.30 ± 0.93	22.64 ± 38.24	50.34	19.73 ± 15.91
	**pCCA**	328	2.94 ± 0.27	173.06 ± 62.25	99.05	0.63 ± 0.73	17.47 ± 33.88	17.57	17.81 ± 16.26
	**GBC**	228	2.78 ± 0.47	137.94 ± 76.6	97.29	0.45 ± 0.67	7.17 ± 20.40	12.67	27.91 ± 18.18
	**dCCA**	185	2.93 ± 0.19	155.78 ± 71.91	100	0.35 ± 0.59	5.67 ± 17.14	8.74	21.30 ± 17.75
	**PDAC**	131	2.89 ± 0.24	177.36 ± 50.45	100	0.34 ± 0.44	3.02 ± 5.60	3.85	n.a.
**Premalignant lesion**	**BilIN**	167	2.99 ± 0.06	240.74 ± 34.73	100	0.88 ± 0.65	34.22 ± 42.22	23.87	22.33 ± 17.07
	**PanIn**	107	2.98 ± 0.24	218.49 ± 42.16	100	0.51 ± 0.79	17.58 ± 38.88	14.56	n.a.
**Non-neoplastic tissue**	**Small bile ducts**	59	2.80 ± 0.34	165.88 ± 71.12	100	2.29 ± 0.59	88.32 ± 56.56	97.96	3.00 ± 5.48
	**Large bile ducts**	42	2.97 ± 0.16	239.47 ± 36.2	100	1.03 ± 0.72	24.02 ± 29.17	34.15	5.49 ± 7.89
	**Perihilar bile ducts**	65	3.00 ± 0	218.40 ± 50.39	100	0.98 ± 0.79	38.70 ± 41.70	33.33	5.82 ± 6.92
	**Gallbladder epithelium**	148	3.00 ± 0.05	179.03 ± 54.39	100	0.47 ± 0.66	19.72 ± 40.23	12.84	8.64 ± 7.73
	**Pancreatic ducts**	92	2.85 ± 0.34	188.45 ± 42.85	100	0.59 ± 0.70	50.58 ± 70.39	8.14	n.a.

^†^ defined as an E-/N-cadherin score of ≥1.5. n.a.: not analyzed/data not available.

## Data Availability

The data are not publicly available due to the data containing information that could compromise the privacy of the research participants. All data included in this study are available upon request from the corresponding author.

## Supplementary Materials

The following supporting information can be downloaded at: https://www.mdpi.com/article/10.3390/cancers14133091/s1, Figure S1: N-cadherin-positive apical secretions of pancreatic ducts; Figure S2: E- and N-cadherin expression in carcinomas of the pancreatobiliary system; Figure S3: Survival analysis of E- and N-cadherin protein expression in iCCAs of our cohort in comparison to CDH1 and 2 mRNA expression in the independent TCGA cohort; Figure S4: the uncropped Western blots; Table S1: patient characteristics of iCCA for the survival analysis (own cohort), separated by high and low N-cadherin H-scores; Table S2: Summary of evaluation of CK7 staining; Table S3: of CA19-9 staining; Table S4: of EMA staining; Table S5: of CDX2 staining; and Table S6: of CK20 staining in the cohort.
