# Supplementary material for: N-Cadherin Distinguishes Intrahepatic Cholangiocarcinoma from Liver Metastases of Ductal Adenocarcinoma of the Pancreas
Source: Cancers (Basel). 2022 Jun 23;14(13):3091. doi: 10.3390/cancers14133091 (PMC9264797; doi:10.3390/cancers14133091)
Supplement: Supplementary file 1 [file cancers-14-03091-s001.zip › cancers-1758375-Supplementary.pdf]

cancers

MDPI

Supplementary Material: N-Cadherin Distinguishes Intrahepatic Cholangiocarcinoma from Liver Metastases of Ductal Adenocarcinoma of the Pancreas

Tiemo S. Gerber, Benjamin Goeppert, Anne Hausen, Hagen R. Witzel, Fabian Bartsch, Mario Schindeldecker, Lisa-Katharina Gröger, Dirk A. Ridder, Oscar Cahyadi, Irene Esposito, Matthias M. Gaida, Peter Schirmacher, Peter R. Galle, Hauke Lang, Wilfried Roth and Beate K. Straub

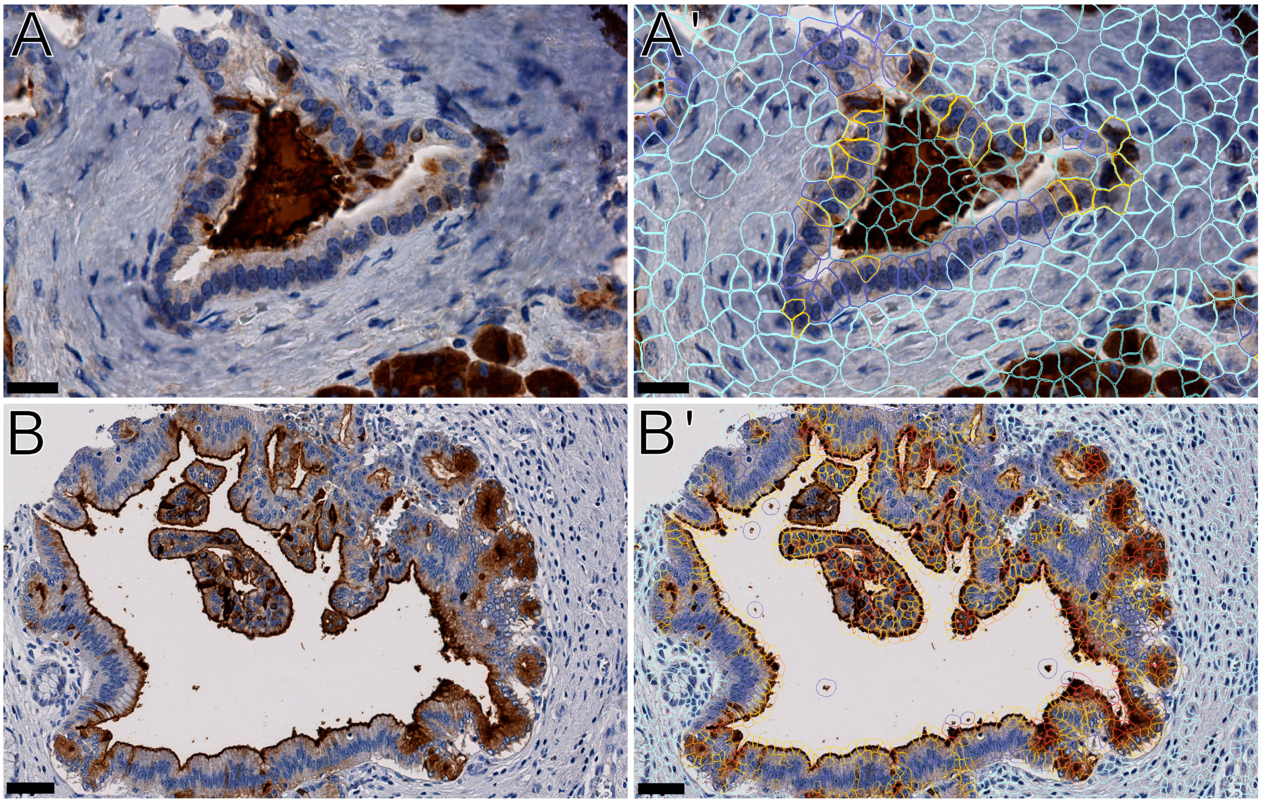

Figure S1. N-cadherin-positive apical secretions of pancreatic ducts.

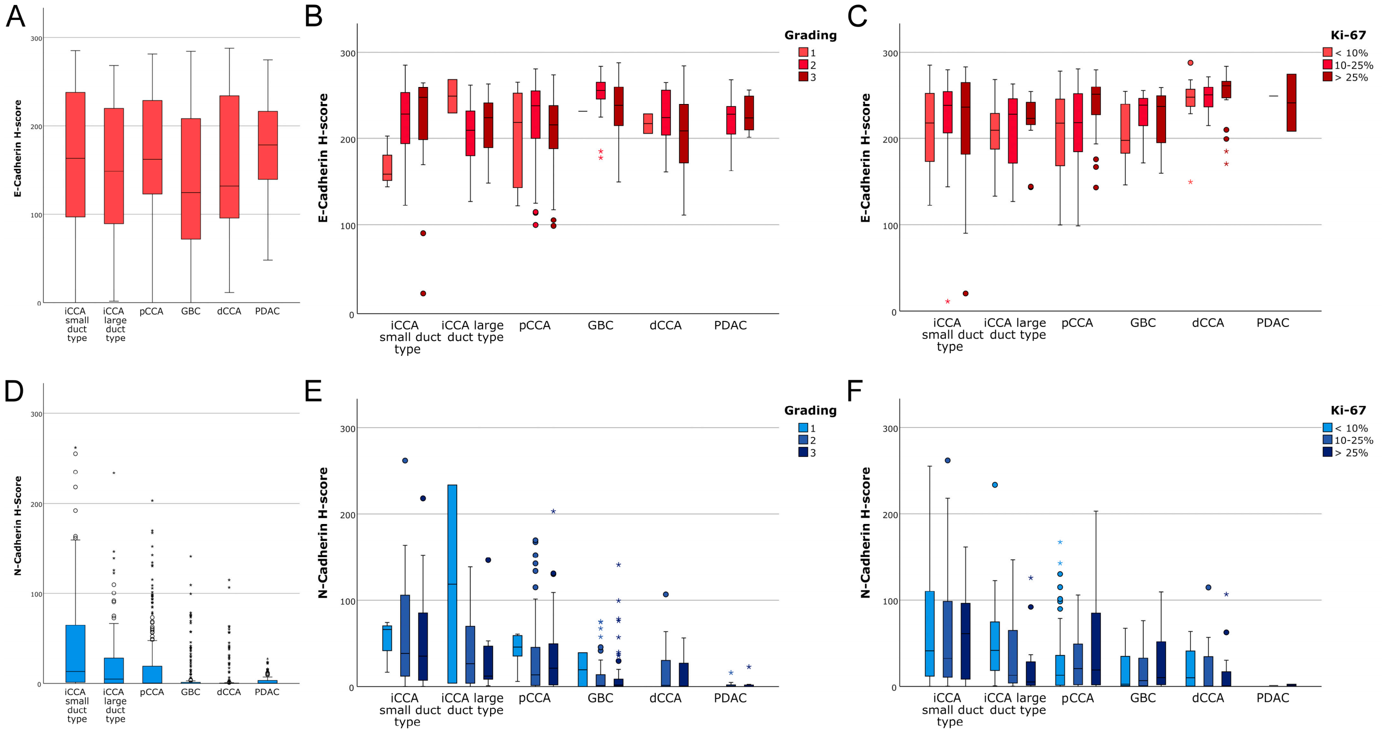

Figure S2. E- and N-cadherin expression in carcinomas of the pancreatobiliary system.

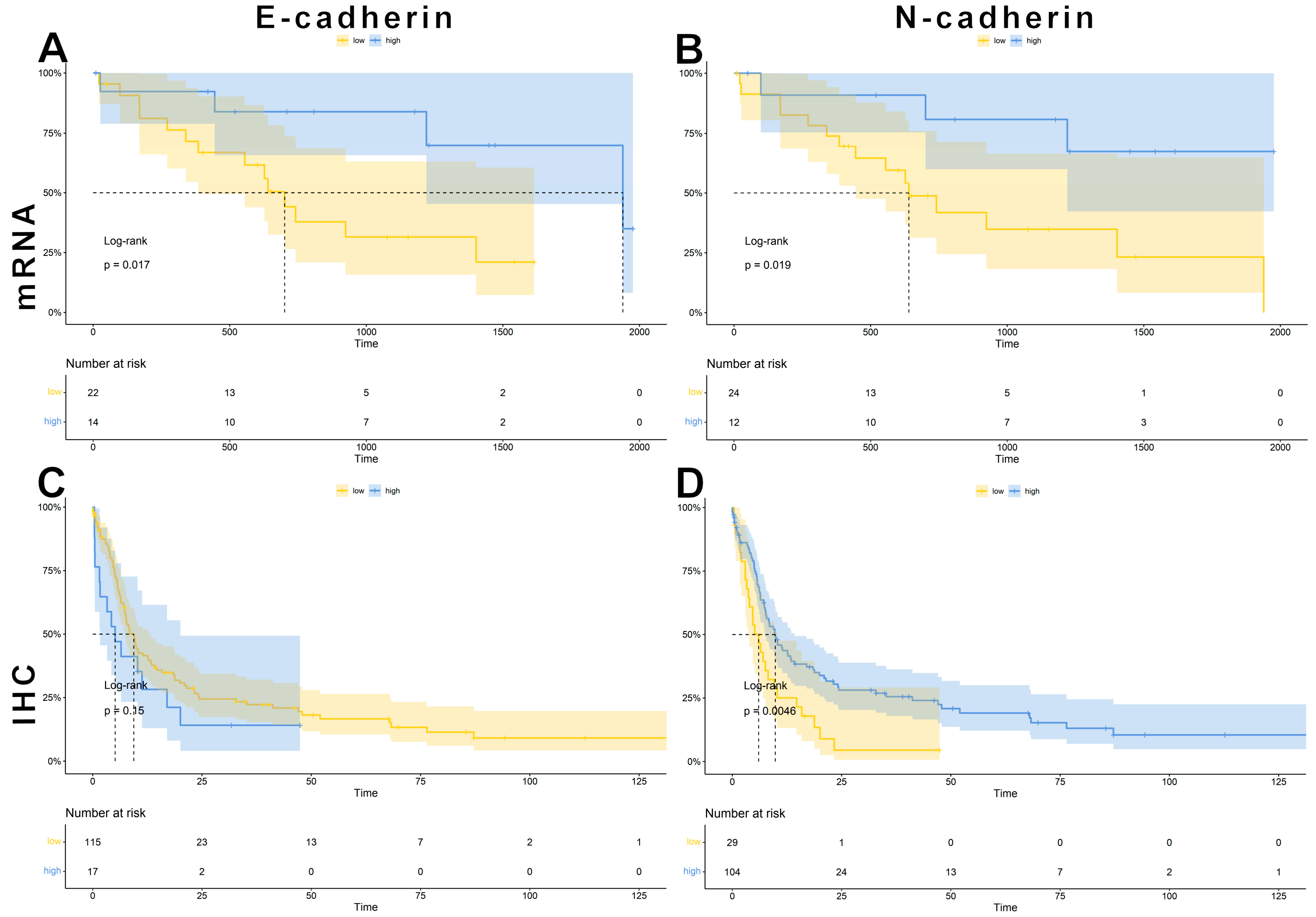

Figure S3. Survival analysis of E- and N-cadherin protein expression in iCCAs of our cohort in comparison to CDH1 and 2 mRNA ex-pression in the independent TCGA cohort.

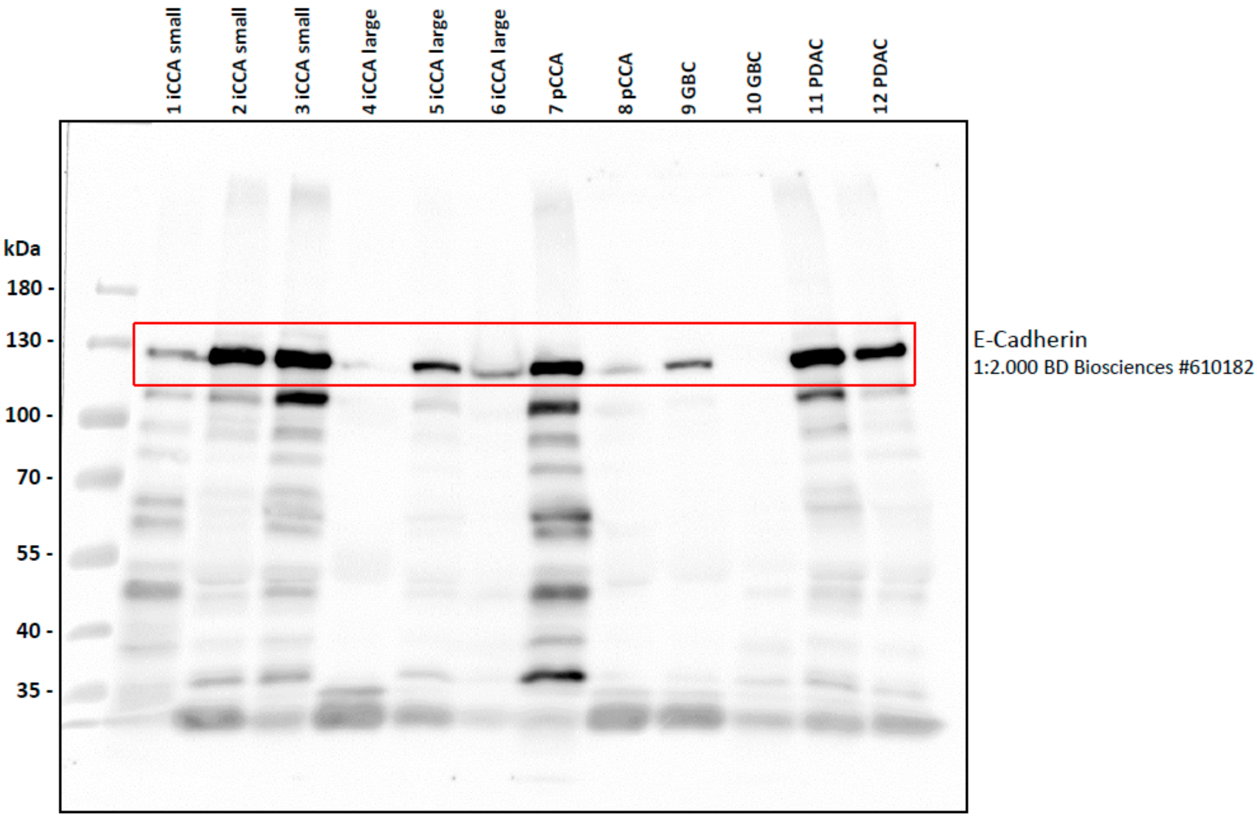
1 iCCA small
2 iCCA small
3 iCCA small
4 iCCA large
5 iCCA large
6 iCCA large
7 pCCA
8 pCCA
9 GBC
10 GBC
11 PDAC
12 PDAC
kDa
180 -
130 -
100 -
70 -
55 -
40 -
35 -
E-Cadherin
1:2.000 BD Biosciences #610182

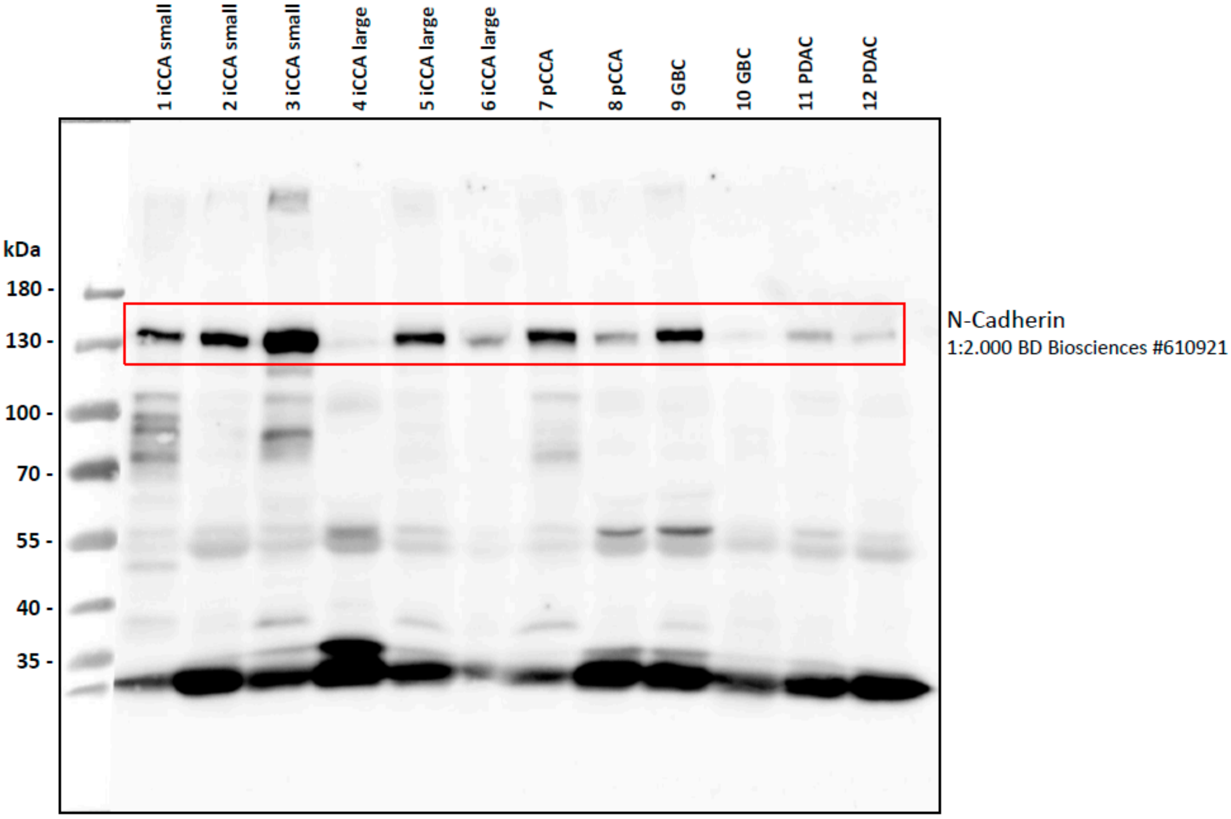
1 iCCA small
2 iCCA small
3 iCCA small
4 iCCA large
5 iCCA large
6 iCCA large
7 pCCA
8 pCCA
9 GBC
10 GBC
11 PDAC
12 PDAC
kDa
180 -
130 -
100 -
70 -
55 -
40 -
35 -
N-Cadherin
1:2.000 BD Biosciences #610921

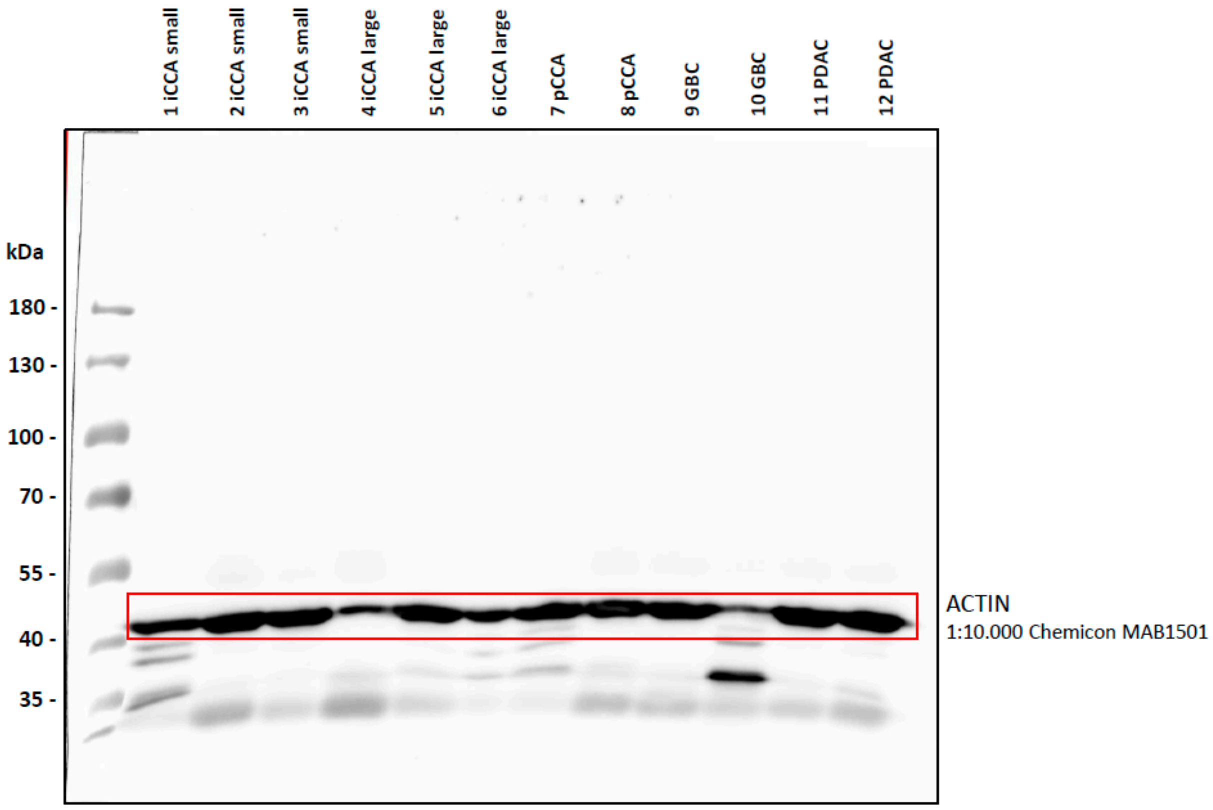

Figure S4. The uncropped Western blots.

Table S1. Patient characteristics of iCCA for the survival analysis (own cohort), separated by high and low N-cadherin H-score (cutoff: 12.16809).

	**High N-cadherin H-score**	**Low N-cadherin H-score**
	***n* = 104**	***n* = 29**
Age †	64.31±10.91	65.75±11.14
Male	62	13
Female	42	16
**E-cadherin**		
Intensity †	2.94±0.17	2.97±0.08
H-score †	209.86±43.01	213.73±42.53
**N-cadherin**		
Intensity †	2.06±0.63	1.04±0.54
H-score †	68.33±47.64	5.36±4.19
**Proliferation** ‡	13.08±9.12	19.80±17.10
**Histology**		
Small duct type	66% (69)	52% (15)
Large duct type	34% (35)	48% (14)
Tumor size †	6.61±3.39	8.46±4.21
pT1	61	15
pT2	30	5
pT3	8	4
pT4	5	5
UICC I	53	9
UICC II	16	4
UICC III	31	11
UICC IV	4	5
pNX	26	4

pN0	52	9
pN1	26	16
pN2	0	0
G1	4	0
G2	78	22
G3	22	7
G4	0	0
L0	91	24
L1	13	5
V0	82	23
V1	22	6
Pn0	81	19
Pn1	23	10
RX	3	1
R0	79	25
R1	22	3

† mean value ± standard deviation. ‡ measured in % immunoreactive tumor cells for Ki-67.

Table S2. Summary of evaluation of CK7 staining in the cohort.

	N	NONE	WEAK	MODERATE	STRONG
**iCCA**		**1.49%**	**4.48%**	**19.4%**	**74.63%**
small duct type	134	2	6	26	100
**iCCA metastases**		**9.68%**	**16.13%**	**22.58%**	**51.61%**
small duct type	31	3	5	7	16
**iCCA**		**2.38%**	**17.86%**	**9.52%**	**70.24%**
large duct type	84	2	15	8	59
**iCCA metastases**		**3.7%**	**0%**	**14.81%**	**81.48%**
large duct type	27	1	0	4	22
**PDAC**		**2.46%**	**2.46%**	**19.67%**	**75.41%**
	122	3	3	24	92
**PDAC metastases**		**3.13%**	**3.13%**	**37.5%**	**56.25%**
	64	2	2	24	36

Table S3. Summary of evaluation of CA19-9 staining in the cohort.

	N	NONE	WEAK	MODERATE	STRONG
**iCCA**		**19.01%**	**41.55%**	**28.17%**	**11.27%**
small duct type	142	27	59	40	16
**iCCA metastases**		**20.69%**	**41.38%**	**31.03%**	**6.9%**
small duct type	29	6	12	9	2
**iCCA**		**10.71%**	**36.9%**	**16.67%**	**35.71%**
large duct type	84	9	31	14	30
**iCCA metastases**		**3.7%**	**33.33%**	**18.52%**	**44.44%**
large duct type	27	1	9	5	12
**PDAC**		**5.88%**	**9.24%**	**12.61%**	**72.27%**
	119	7	11	15	86
**PDAC metastases**		**7.69%**	**12.31%**	**4.62%**	**75.38%**
	65	5	8	3	49

Table S4. Summary of evaluation of the EMA staining in the cohort.

	N	NONE	WEAK	MODERATE	STRONG
**iCCA**		**4.23%**	**31.69%**	**40.14%**	**23.94%**
small duct type	142	6	45	57	34
**iCCA metastases**		**9.68%**	**22.58%**	**54.84%**	**12.9%**
small duct type	31	3	7	17	4
**iCCA**		**0%**	**22.62%**	**33.33%**	**44.05%**
large duct type	84	0	19	28	37
**iCCA metastases**		**0%**	**7.41%**	**48.15%**	**44.44%**
large duct type	27	0	2	13	12
**PDAC**		**0.83%**	**11.57%**	**37.19%**	**50.41%**
	121	1	14	45	61
**PDAC metastases**		**0%**	**13.85%**	**47.69%**	**38.46%**
	65	0	9	31	25

Table S5. Summary of the evaluation of the CDX2 staining in the cohort.

	N	NONE	WEAK	MODERATE	STRONG
**iCCA**		**99.3%**	**0%**	**0%**	**0.7%**
small duct type	142	141	0	0	1
**iCCA metastases**		**96.77%**	**0%**	**0%**	**3.23%**
small duct type	31	30	0	0	1
**iCCA**		**94.05%**	**4.76%**	**0%**	**1.19%**
large duct type	84	79	4	0	1
**iCCA metastases**		**85.71%**	**14.29%**	**0%**	**0%**
large duct type	28	24	4	0	0
**PDAC**		**97.54%**	**1.64%**	**0.82%**	**0%**
	122	119	2	1	0
**PDAC metastases**		**95.38%**	**3.08%**	**1.54%**	**0%**
	65	62	2	1	0

Table S6. Summary of the evaluation of the CK20 staining in the cohort.

	N	NONE	WEAK	MODERATE	STRONG
**iCCA**		**85.21%**	**11.97%**	**0.7%**	**2.11%**
small duct type	142	121	17	1	3
**iCCA metastases**		**83.87%**	**12.9%**	**3.23%**	**0%**
small duct type	31	26	4	1	0
**iCCA**		**71.43%**	**14.29%**	**6.49%**	**7.79%**
large duct type	77	55	11	5	6
**iCCA metastases**		**42.86%**	**50%**	**7.14%**	**0%**
large duct type	28	12	14	2	0
**PDAC**		**72.95**	**18.03%**	**4.92%**	**4.1%**
	122	89	22	6	5
**PDAC metastases**		**73.85%**	**20%**	**4.62%**	**1.54%**
	65	48	13	3	1
